# Why are we all not ten feet tall (or point masses)?

**DOI:** 10.1098/rspb.2026.1885

**Published:** 2026-11-11

**Authors:** Charles F. Baer, Henrique Teotónio

**Affiliations:** ^1^ Department of Biology and University of Florida Genetics Institute, University of Florida, Gainesville, FL, USA; ^2^ Institut de Biologie de l’École Normale Supérieure, ENS, CNRS UMR8197, Inserm U1024, PSL Research University, Paris, France

**Keywords:** quantitative genetics, *Drosophila*, mutation, experimental evolution

Few sayings are repeated with such confidence, or scrutinized so rarely, as ‘size matters’. There is abundant evidence that many organisms experience directional selection for increased body size. In fact, inbreeding depression is so called because inbred individuals are typically smaller than their outbred relatives, the reason being that deleterious recessive alleles tend to reduce body size when homozygous [1,2]. And yet, as our title indicates, something must ultimately limit the evolution of body size.

Mutation is the ultimate source of genetic variation, and in any evolutionary scenario, mutation gets a vote. One possibility is that body size increases until mutations that cause a further increase in size have such large deleterious pleiotropic effects that the population reaches mutation-selection balance. This possibility gains credence from evidence of selective breeding in livestock, in which strong artificial selection to increase elements of size has produced breeds that are severely compromised with respect to fitness-related traits (e.g. mating ability, immune function) [3]. A second possibility is that body size is, in fact, under stabilizing selection at some intermediate optimum, but that—for whatever reason—mutations tend to decrease body size, leading to apparent directional selection upward [4].

A third possibility is that there is epistasis, where the effects of mutation depend on the underlying genotype. This possibility, which is not mutually exclusive with the selection-based explanations, is that the distribution of mutational effects (DMEs) itself depends on the underlying genotype (i.e. there is epistasis) in such a way that a mutation that affects body size has a smaller effect in a more extreme individual. If so, as size increases, the DME hits diminishing returns (figure 1A) and the heritable variation for increased size approaches zero. As size decreases, again the DME hits diminishing returns and there will be little heritable variation, with the implication being that genotypes of intermediate size should have more heritable variation ( figure 1A). Conversely, if effects are magnified in more extreme genetic backgrounds (e.g. large begets larger), individuals with extreme large and small genotypes will increasingly diverge until they bump up against the boundary imposed by pleiotropic selection (figure 1B).

**Figure 1.**
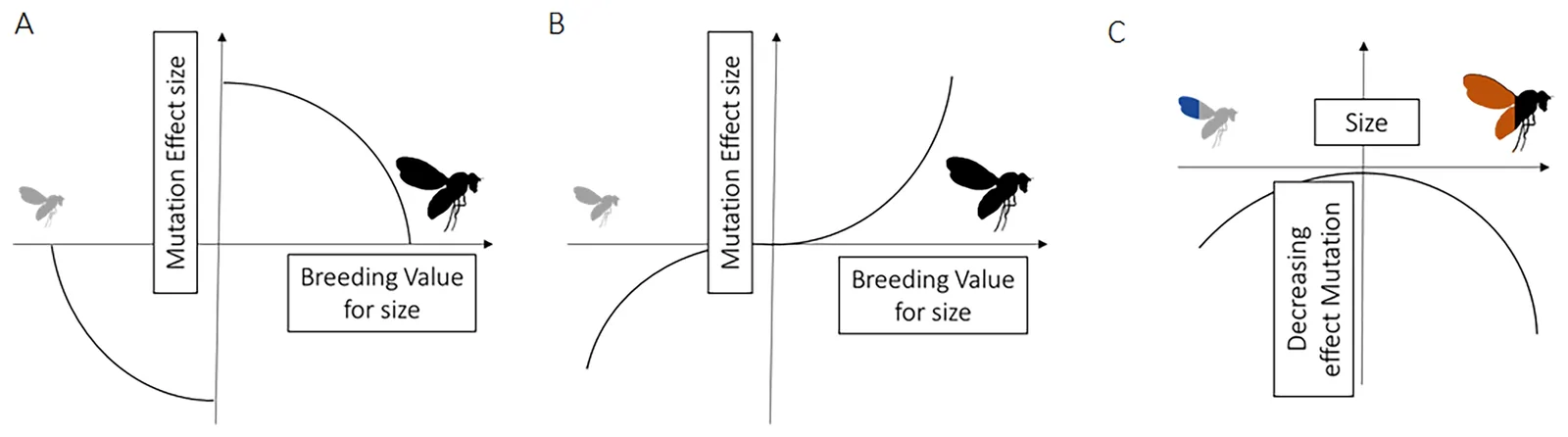
(A) Expected DMEs for body size under diminishing-returns (negative) epistasis. Body size is assumed to be a polygenic trait in which the phenotypic effect of a new mutation depends on the genetic background. Mutations have their largest expected effects in individuals with intermediate breeding values, whereas their effects decline progressively in individuals with more extreme breeding values (either larger or smaller than the population mean). Breeding values are defined relative to the focal population, so intermediate genotypes are centred at the origin. Mutations may increase or decrease body size, and these expectations assume that the mutation rate is independent of genotype. (B) Similarly, but with synergistic (positive) epistasis. The phenotypic effects of new mutations increase with the magnitude of breeding value, such that mutations have the largest expected effects in the most extreme genotypes and the smallest effects in individuals with intermediate breeding values. (C) King & Mcguigan's MA experiments suggest synergistic epistasis for body size in *D. serrata*. Mutation effects are smallest in genotypes with intermediate breeding values and become increasingly negative as breeding value departs from the population mean, with the major reductions in size occurring in the largest genotypes (blue versus red).

To test hypotheses about genetic variation, it is necessary to separate the effects of mutation from those of natural selection. That can be done by means of a ‘mutation accumulation’ (MA) experiment, in which replicate populations derived from a common (ideally genetically homogeneous) ancestor are propagated at very small genetic effective population size (*N_e_*), such that all but the most strongly deleterious mutations are ‘invisible’ to natural selection (*s* ≳1/2*N_e_*; [5]). The observable output from an MA experiment is the change in the trait mean over time (the mutational bias, Δ*M*) and the increase in the genetic variance contributed by new mutations (the mutational variance, *V_M_*). Δ*M* and *V_M_* are composite functions of the genomic mutation rate (*U*) and the average mutational effect, *ɑ*; Δ*M* = *Uɑ* and *V_M_* = *Uɑ*^2^ [6,7].

To address the relationship between initial (heritable) phenotype and the subsequent phenotypic effects of new mutations, King & McGuigan investigated the effects of accumulated mutations on wing size, a surrogate for body size, in the fruit fly *Drosophila serrata* [8]. They started with five unrelated inbred strains from the same wild population from Brisbane, Australia (strains A–E, arranged from smallest to largest breeding value for wing size; range approximately equal to 10%; one of which, the second-smallest strain, had very low fitness and was excluded from the analysis). The first thing to note is that this was a very large, well-powered experiment. The number of MA lines in each strain (approx. 64) is typical of studies that investigate only one starting genotype, let alone five. What is more, each MA line was measured at each generation (32 generations); many such studies only assay the phenotype a few times or even only at the culmination of the MA phase.

The results were interesting (‘may you live in interesting times’). Of the four remaining strains, the smallest (A) and the two largest (D, E) showed a significant mutational bias towards smaller size. This result is unsurprising; MA studies with a variety of organisms have consistently reported that deleterious mutations tend to reduce body size. However, the bias was stronger in the two large strains than in the smallest strain. The smallest (A) and largest (E) strains had the largest mutational variances, with the second-largest strain (D) intermediate. The second-smallest strain (C) was closest to the midpoint of the size distribution and had no significant mutational variance (and thus no mutational bias). The random environmental variance (*V_E_*) was significantly lower in strain C than in the other strains.

Different evolutionary responses (i.e. Δ*M* and *V_M_*) under the same demographic and environmental conditions mean that the average mutational effect (*ɑ*) and/or the mutation rate (*U*) must differ among strains. Assuming the mutation rate is constant (it may not be), it means there is epistasis. On a large genetic background (strains D and E), the average mutation decreases wing size by more than it does on a small genetic background (strain A). However, on a medium genetic background (strain C), mutations apparently have no effect on wing size (figure 1C). And ‘no effect’ means just that; if some mutations increased size and some mutations decreased size, we would expect variation among MA lines and *V_M_* would be non-zero.

So, what to make of these results? King & McGuigan posit that (i) wing size is under stabilizing selection at an intermediate optimum, (ii) that in the vicinity of the optimum the phenotype is buffered (‘canalized’ or ‘robust’; [9]) against the effects of both new mutations (*V_M_* approx. equal to 0) and random environmental perturbations (residual component of trait variance, *V_E_*, is small), (iii) that as wing size evolves away from the optimum, the phenotype becomes de-canalized, revealing (iv) the consistent downward bias of deleterious mutations. Implicit in that scenario is the pleiotropic mutation-selection balance argument noted above, but at the small end; as flies get progressively smaller, at some point a further reduction in size becomes so deleterious that size-reducing mutations will not accumulate even under MA conditions.

What then ultimately limits the evolution of body size? King & McGuigan conclude from their experiments that both selection and mutation constrain body size evolution, consistent with all three scenarios outlined above. Stabilizing selection around an intermediate optimum may generate canalized phenotypes and contribute to evolutionary stasis, while a mutational bias towards decreased size could be offset in small-bodied individuals by directional selection for increased size. By contrast, for large-bodied individuals, directional selection towards the optimal trait size (i.e. for intermediate-sized individuals) would reinforce mutational bias.

It is somewhat puzzling that body size in animals is generally thought to have a predominantly additive genetic architecture [10], with evidence from *Drosophila* and humans, to take two well-studied examples, indicating that this variation is highly polygenic [11,12]. King & McGuigan find clear evidence for epistasis. In line with previous results from the same lab studying the same *D. serrata* population [13], one explanation that could reconcile these observations is that mutations with strong epistatic effects on size are sufficiently deleterious that they are largely eliminated by purifying selection, leaving predominantly additive genetic variation segregating in natural populations. The finding that individuals at the trait optima are more canalized because of stabilizing selection will surely deserve more discussion. Theoretical models predict that there can only be very weak (second-order) selection leading to the evolution of canalization [14,15].

King & McGuigan's experiments uniquely link standing genetic variation, mutational effects and selection, and they raise the intriguing possibility that similar processes may underlie the evolution of body size in other populations and species. It is worth asking whether other traits, especially those only loosely connected to fitness, such as molecular phenotypes (mRNA, proteins and metabolites), undergo similar evolution.

## Data Availability

This article has no additional data.
